# Papillary Carcinoma Arising From Thyroglossal Duct Cysts: A Case Series and Management Strategies

**DOI:** 10.7759/cureus.79394

**Published:** 2025-02-21

**Authors:** Adhithya N Balaji, Balaji Balasubramanian, Amritaa Thalla, Yogesh Gohil, Panna Shetty

**Affiliations:** 1 Internal Medicine, Università Cattolica Del Sacro Cuore, Rome, ITA; 2 Surgery and Oncology, New Medical Centre (NMC) Specialty Hospital, Abu Dhabi, ARE; 3 Internal Medicine, Maheshwara Medial College and Hospital, Isnapur, IND; 4 Radiology, New Medical Centre (NMC) Specialty Hospital, Abu Dhabi, ARE; 5 Pathology, New Medical Centre (NMC) Royal Hospital, Khalifa City, Abu Dhabi, ARE

**Keywords:** papillary carcinoma, sistrunk procedure, thyroglossal duct cyst, thyroidectomy, thyroid neoplasm

## Abstract

Papillary carcinoma arising from thyroglossal duct cysts is a rare entity, most often detected after surgery. Despite its excellent prognosis, management remains controversial. In this report, we present three cases of papillary carcinoma in thyroglossal duct cysts. In two of the three cases, malignancy was identified in both the thyroglossal duct cyst and the thyroid gland at the time of presentation. In contrast, the third case was diagnosed with malignancy only after surgery for the thyroglossal duct cyst, ultimately necessitating a total thyroidectomy.

A high degree of suspicion and clinical awareness are required during evaluation of this rare situation which is otherwise usually a benign lesion. For low-risk patients with papillary carcinoma involving only the thyroglossal duct cyst, the Sistrunk procedure (resection of the thyroglossal duct cyst) is adequate. For high-risk cases of papillary carcinoma of the thyroglossal duct cyst with an associated malignancy of the thyroid gland, total thyroidectomy, central compartment nodal dissection, and radioactive iodine ablation are needed.

## Introduction

Thyroglossal duct cysts are one of the most common anomalies of thyroid gland development, accounting for the majority of midline neck swellings in childhood [1]. The incidence of midline neck masses diagnosed as thyroglossal duct cysts is about 70-75% in childhood and approximately 7% in adults [2]. Notably, thyroid carcinoma develops from thyroglossal duct cysts in only 1% of cases [3]. There are very few studies with a significant number of cases due to the rarity of the disease [2,4]. Currently, there are approximately 300 published cases of thyroglossal duct cysts with papillary carcinoma, most of them being case reports [5]. Papillary carcinoma is the most common malignancy associated with thyroglossal duct cysts, accounting for 75%-83% of cases [5]. Other tumor types include mixed papillary-follicular carcinoma (7%), squamous cell carcinoma (5%), and follicular carcinoma (1.7%), with rarer reports of Hürthle cell carcinoma and anaplastic carcinoma (0.9%). Squamous cell carcinoma, which constitutes about 5% of these tumors, is associated with a poorer prognosis [6]. Notably, medullary carcinoma has not been documented in thyroglossal duct cyst malignancy in medical literature [2]. Cervical nodal metastasis occurs in approximately 8%-11.3% of cases [4]. These de novo tumors can exhibit biological behavior similar to their primary gland counterparts.

## Case presentation

Case 1

**Figure 1:**
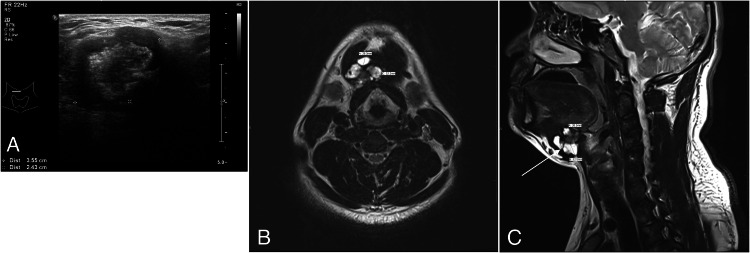
(A) US transverse image through the right paramedian thyrohyoid region shows a well-defined heterogeneous lesion with a peripheral thick hypoechoic wall and internal echogenic content. (B) Axial T2-weighted MRI of the neck shows a well-defined lesion with mixed signal intensity in the right paramedian region of the upper neck, anterior to thyrohyoid region with involvement of floor of mouth. It shows internal cystic areas, thick septations as well as solid hypodense components. (C) Sagittal T2-weighted MRI of the neck shows a well-defined mixed signal intensity lesion (arrow) in the anterior thyrohyoid region of the upper neck with involvement of floor of mouth. It shows internal cystic areas, thick septations, and solid hypodense components. US: Ultrasound, MRI: Magnetic Resonance Imaging

A 32-year-old man presented with a painless mass in the upper neck noticed one month ago. On clinical examination, a 4 cm mass was palpable in the midline of the upper neck, above the thyroid cartilage prominence. The mass moved with tongue protrusion, thereby suggesting a thyroglossal duct cyst. The thyroid gland was not palpable, and no gross lymph nodes were felt in the lateral neck. Oral cavity examination was unremarkable. The patient had no history of radiation in the past. Posterior tongue and vocal cord examination was normal. The thyroid profile was within normal limits. Imaging with an ultrasound (US) scan showed a well-defined heterogeneous lesion with a peripheral thick hypoechoic wall and internal echogenic content (Figure 1A). Further evaluation with a magnetic resonance imaging (MRI) scan showed a lobulated, mixed intensity mass lesion, of approximate size of 3.5 cm x 2.8cm x 4 cm, in the midline of the upper neck extending towards the right side, anterior to hyoid bone, thyrohyoid membrane and upper thyroid cartilage. Adjacent muscle invasion was suspected. The lesion showed an internal mixed (hypo and hyperintense) signal, areas of calcifications, and probable post-contrast enhancement (Figures 1B, 1C). 

**Figure 2:**
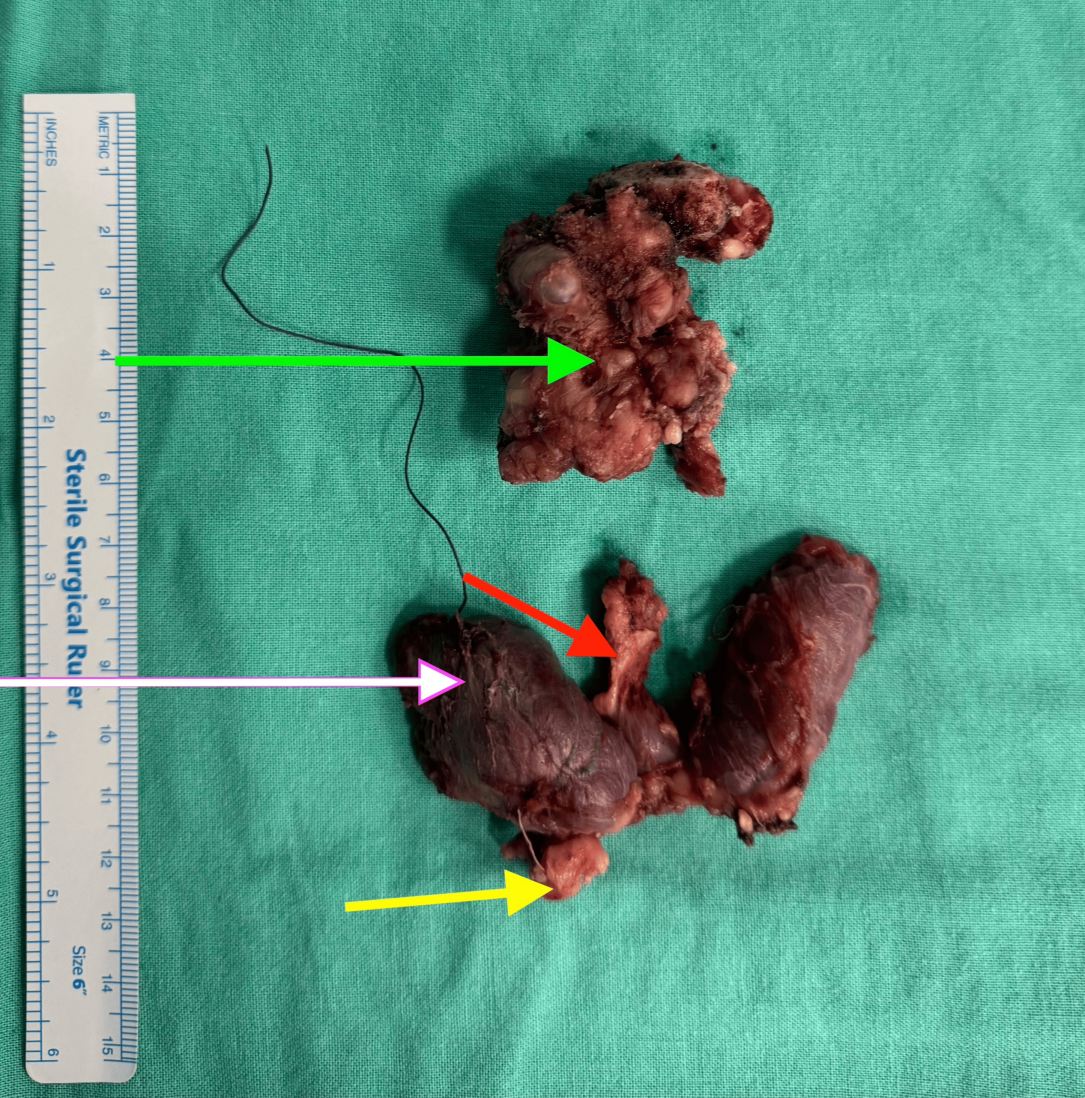
Surgical specimen showing the right lobe of the thyroid (white arrow), pyramidal lobe (red arrow), paratracheal lymph node (yellow arrow), and the primary tumor (green arrow).

Clinicoradiologically, it was suspected to be a thyroglossal duct cyst with possible malignancy due to suspected muscle invasion. Ultrasound-guided fine needle aspiration cytology (US-guided FNAC) was done for the cystic lesion which was reported as papillary carcinoma. The patient was subsequently managed surgically with a total thyroidectomy and thyroglossal duct cyst resection (Sistrunk procedure) with central compartment nodal dissection. During surgery, the body of the hyoid was excised along with the specimen (Figure 2).

**Figure 3:**
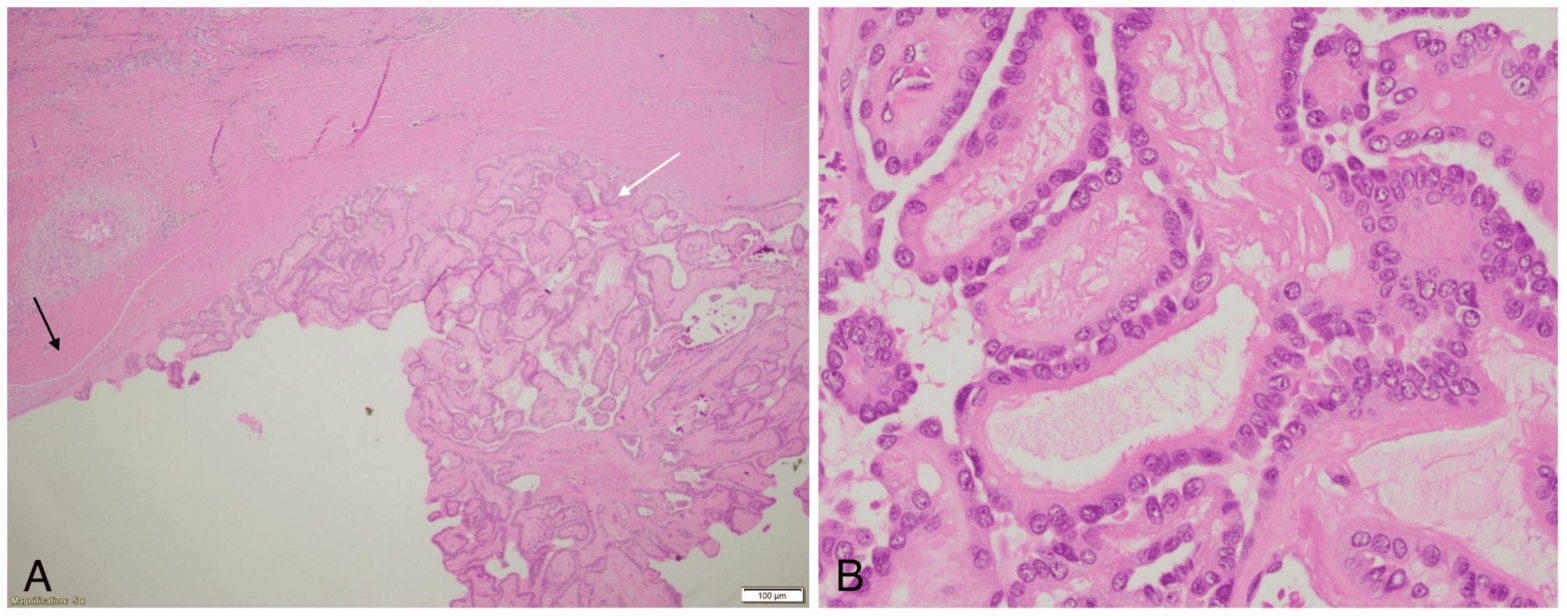
Histopathology of the specimen stained with H&E showing (A) papillary tumor in the cyst and (B) slide highlighting the nuclear features of the papillary tumor with nuclear crowding, grooves, nucleoli, and inclusions. H&E: Hematoxylin and eosin, (A) 4x magnification, (B) 40x magnification, black arrow: cyst wall, white arrow: tumor

There was minimal soft tissue invasion into the adjacent muscles around the thyroglossal duct cyst. The tumor was completely resected, and histopathological analysis revealed multifocal papillary carcinoma involving the thyroglossal duct cyst and both thyroid lobes (Figure 3).

Metastasis was identified in 1 out of 10 lymph nodes, and invasion into the strap muscles was noted. The pathological staging was pT3bN1aMx. The patient underwent radioiodine (RAI) ablation with 100 mCi and has been maintained on a suppressive dose of levothyroxine (150 mcg once daily). Follow-up testing showed a thyroglobulin level of 0.1 ng/mL, and the patient has remained disease-free for the past one year.

Case 2

**Figure 4:**
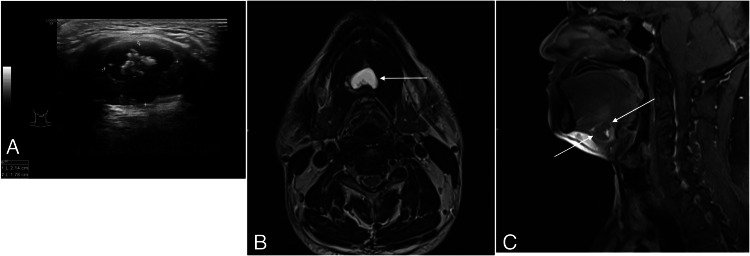
(A) US transverse image through the right mid thyrohyoid region shows a well-defined complex cystic lesion with a thick irregular wall and internal bright echogenic content which shows posterior shadowing. (B) Axial T2-weighted MRI of the neck shows a well-defined complex cystic lesion in the mid upper neck anterior to the thyrohyoid region with involvement of floor of mouth. (C) Sagittal T1 fat saturated post contrast (gadolinium) MRI of the neck shows a complex cystic lesion in the upper neck anterior to the thyrohyoid region with involvement of the floor of the mouth. It shows an enhancing thick irregular wall and a posteriorly situated enhancing nodule. US: Ultrasound, MRI: Magnetic Resonance Imaging

A 48-year-old man presented with a painless swelling in the upper neck. Clinical examination revealed a firm, 2.5 cm swelling in the region of the hyoid bone that moved with tongue protrusion, consistent with a thyroglossal duct cyst. US scan showed a lobulated, complex cystic mass in the midline of the neck at the level of the hyoid bone. The mass was located near the strap muscles, bulging into the overlying subcutaneous tissue, and contained an eccentric internal solid component with multiple punctate echogenic foci (Figure 4A). Additionally, a TR 3 nodule was identified in the left lobe of the thyroid gland. These findings raised suspicion of malignancy in the thyroglossal duct cyst. MRI revealed a complex cystic lesion in the upper neck, anterior to the thyrohyoid region, with involvement of the floor of the mouth. The lesion exhibited an enhancing, thick, irregular wall and a posteriorly situated enhancing nodule (Figures 4B, 4C).

**Figure 5:**
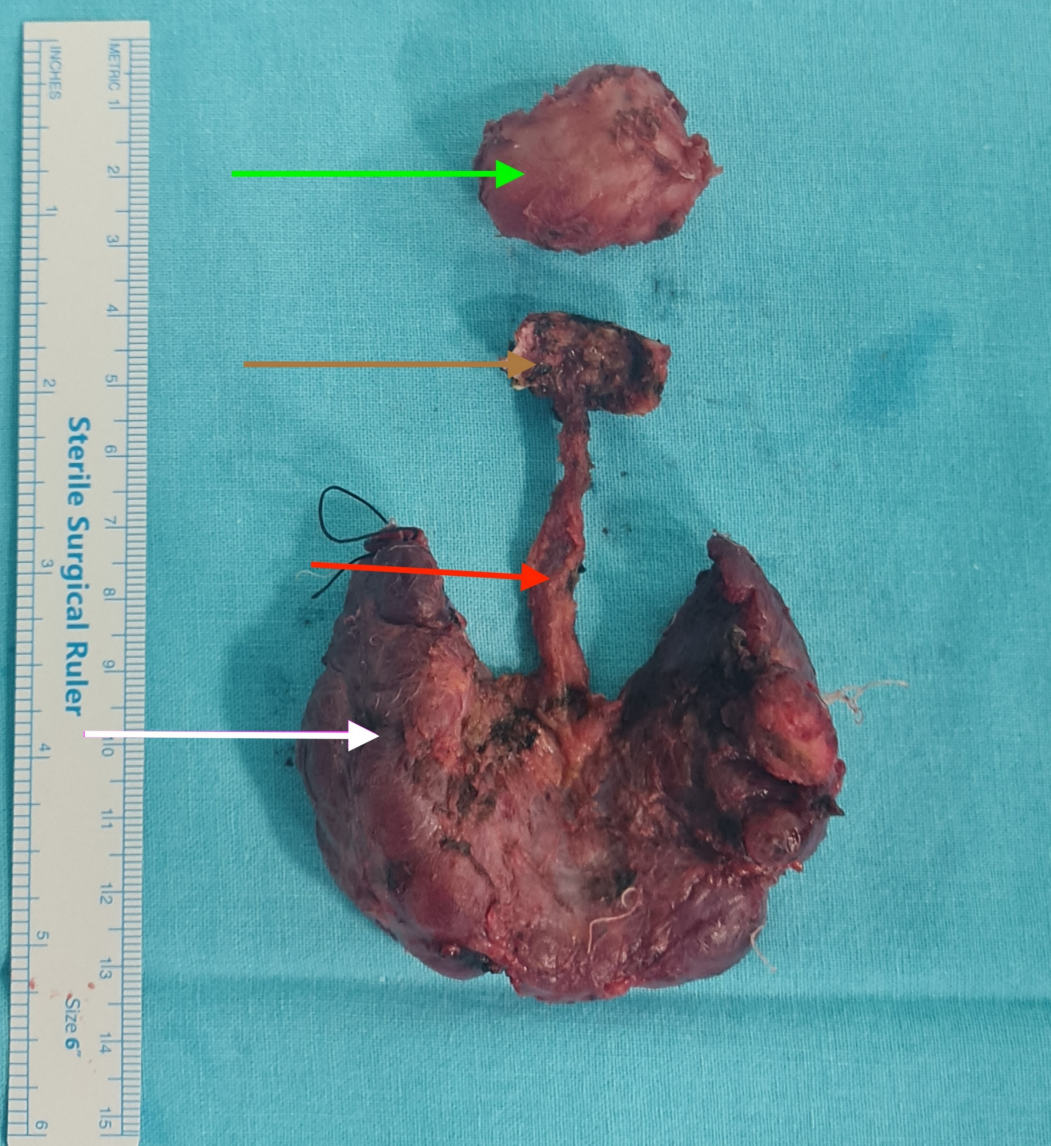
Surgical specimen showing the right lobe of the thyroid (white arrow), the pyramidal lobe (red arrow), the hyoid bone (brown arrow), and the primary tumor (green arrow).

US-guided FNAC from both the thyroglossal duct cyst and the left-sided thyroid nodule was classified as Bethesda category VI, consistent with papillary carcinoma. The patient subsequently underwent total thyroidectomy, excision of the thyroglossal duct cyst with the hyoid bone (Sistrunk procedure), and central compartment nodal dissection (Figure 5).

**Figure 6:**
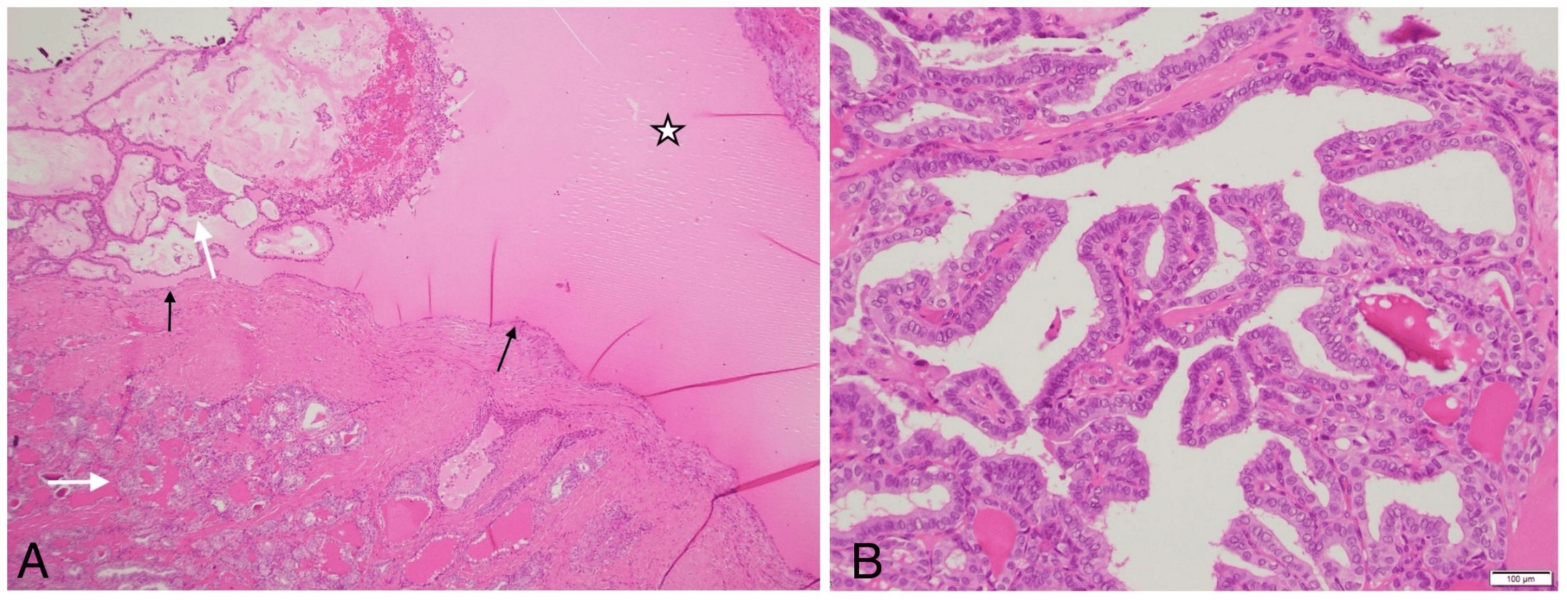
Histopathology of the specimen stained with H&E showing (A) papillary tumor in the cyst, and (B) slide showing the papillary tumor in the cyst wall, highlighting the nuclear features of the tumor with nuclear crowding, grooves, nucleoli and inclusions. H&E: Hematoxylin and eosin, (A) 4x magnification, (B) 40x magnification, black arrows: cyst wall, white arrows: tumor in the cyst wall as well as in the cyst lumen. Cyst fluid is seen highlighted by a white star.

Histopathological examination confirmed multifocal papillary carcinoma involving both thyroid lobes and the thyroglossal duct cyst (Figure 6). There was no extrathyroidal extension, and none of the 9 lymph nodes examined showed metastases. The thyroglossal duct cyst containing papillary carcinoma measured 3 cm at its largest dimension, with a final pathological staging of pT2N0M0.

Postoperatively, the patient received 100 mCi of RAI ablation. He has been disease-free for two years and remains on follow-up. His current thyroglobulin level is 0.1 ng/mL, and he is maintained on a suppressive dose of levothyroxine (125 mcg once daily).

Case 3

**Figure 7:**
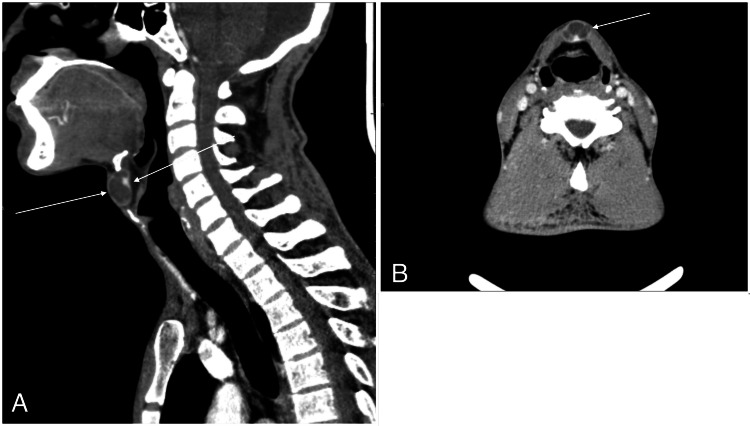
(A) Sagittal post contrast CT scan shows a complex cystic lesion situated in the upper anterior neck, in the midline, between the hyoid bone and the thyroid cartilage. It shows a thin enhancing wall, and a small enhancing nodule situated posteriorly. (B) Axial post contrast CT scan shows a complex cystic lesion situated in the anterior mid upper neck, with a thin enhancing wall and a small enhancing nodule situated posteriorly. CT: Computed tomography

A 50-year-old man presented with a painless swelling in the upper neck. The swelling was midline and moved with tongue protrusion, consistent with a thyroglossal duct cyst. Thyroid function tests were within normal limits. Imaging with US and computed tomography (CT) scans revealed a complex cystic lesion in the anterior upper midline neck, located between the hyoid bone and the thyroid cartilage (Figure 7). The lesion demonstrated a thin enhancing wall with a small posteriorly situated enhancing nodule. The thyroid gland displayed a small, benign-appearing subcentimeter nodule. As there was no suspicion of malignancy in preoperative imaging, FNAC was not done.

The patient underwent a classical Sistrunk procedure for the thyroglossal duct cyst. Histopathological examination revealed papillary carcinoma within the cyst, measuring 1.6 cm in its greatest dimension. A follow-up US scan identified a small paratracheal lymph node with questionable significance and several subcentimeter nodules in both thyroid lobes.

The case was reviewed by the multidisciplinary tumor board, where it was determined that, given the patient’s age (over 45 years) and the radiological findings, a total thyroidectomy would be appropriate. The patient subsequently underwent total thyroidectomy and central compartment nodal dissection. Histopathological examination revealed two microscopic foci of papillary carcinoma in the thyroid, measuring 6 mm and 7 mm, respectively. One of the nine dissected lymph nodes showed microscopic metastasis. The pathological staging was pT1N1aM0.

The patient received 100 mCi of RAI ablation postoperatively. He has been maintained on a suppressive dose of levothyroxine (125 mcg once daily) and has been disease-free with a thyroglobulin level of less than 0.04 ng/mL. He has been under follow-up at our center for 12 years without evidence of recurrence.

## Discussion

Embryology

During embryogenesis, the thyroid gland originates from an invagination at the foramen cecum located at the base of the tongue, around the third or fourth week of gestation. By the seventh week, this tissue descends through or behind the hyoid bone to its final midline position in the neck, anterior to the trachea. Typically, the thyroglossal duct, which facilitates this migration begins to regress by the fifth week and is completely obliterated by the eighth week of gestation. Failure of this duct to obliterate can result in the formation of a thyroglossal duct cyst anywhere along its descent path, a condition observed in approximately 7% of the population [1]. 

The first case of thyroid carcinoma arising from a thyroglossal duct remnant was reported by Brentano in 1911 [7]. The thyroglossal duct cysts can be found along the neck midline in the thyrohyoid (60%), suprahyoid (2%), suprasternal (13%), and intralingual (25%) areas.

Theory of origin

There are two theories postulated to explain the origin of papillary carcinoma in thyroglossal duct cysts [1,8]. Rossi et al. [9] proposed a de novo origin, suggesting that the malignancy arises independently within the thyroglossal duct cyst. The majority of the thyroglossal duct cyst carcinomas develop as a primary malignancy from a thyroid remnant. Baglam et al. [10], however, argued that thyroglossal duct cyst carcinoma may represent a metastatic focus from an occult thyroid tumor. 

Widstrom et al. [11] have defined the following criteria for primary thyroglossal duct malignancies: (i) The carcinoma should be in the wall of the thyroglossal duct cyst; (ii) The thyroglossal duct cyst carcinoma must be differentiated from a cystic lymph node metastasis by histologic demonstration of a squamous or columnar epithelial lining and normal thyroid follicles in the wall of the cyst; (iii) There should be no malignancy in the thyroid gland or any other possible primary site.

Joseph and Komorovski [12] proposed the following criteria for the unequivocal diagnosis of primary thyroglossal duct carcinoma: (i) The finding of a thyroglossal remnant that can be distinguished from a cystic lymph node metastasis by a columnar or squamous epithelial lining; (ii) The presence of a few normal thyroid follicle nests on the cyst wall; (iii) The presence of a normal thyroid gland. 

Controversy exists regarding the origin of thyroid carcinomas occurring within thyroglossal duct cysts as well as the criteria to define primary thyroglossal duct cyst carcinoma. Whether they are a result of metastasis from primary thyroid cancers or arise de novo within the cyst is not yet settled. 

Clinical presentation

Papillary carcinoma arising from thyroglossal duct cysts is more commonly observed in adults, with a female: male ratio of 2.3:1, typically occurring in the third or fourth decade of life [7]. Clinically, it most often presents as an asymptomatic midline neck mass. Intralaryngeal extension is extremely rare and may manifest with symptoms such as dysphonia and dysphagia. The average size of the cyst is about 2 to 4 cm and is usually located at or below the level of the hyoid bone [13]. Malignancy should be suspected if the cyst is hard, fixed, irregular, or exhibits sudden enlargement accompanied by nodal disease [2].

The diagnostic workup begins with clinical examination and US evaluation. One of the largest case series of thyroglossal duct cysts [14], comprising 55 cases, analyzed the radiological characteristics of these lesions. Usually, a thyroglossal duct cyst is diagnosed by US features. On a US scan, a benign thyroglossal cyst can be anechoic, homogenously hypoechoic, homogenously hyperechoic (pseudo‐solid), or heterogeneous in appearance. Malignancy within the thyroglossal duct cyst is suspected when a mural lesion appears within the cyst, sometimes accompanied by microcalcifications or evidence of tumor invasion into the cyst wall.

Role of FNAC

FNAC under US guidance is performed when there is a suspicion of papillary carcinoma in a thyroglossal duct cyst. It plays a crucial role in assessing the extent of the disease and in treatment planning [15]. 

The incidence of papillary carcinoma in thyroglossal cysts is approximately 1%. Given its rarity, the use of FNAC should be judicious and guided by radiological findings. In a series of 12 cases reported by Heshmati et al. [16], 33% of cases demonstrated carcinoma involving both the thyroglossal duct cyst and the thyroid gland. In case of suspicious features on a US scan, such as solid components or wall thickenings, a contrast-enhanced CT or MRI may provide additional diagnostic clarity. 

In our case series, two out of three cases had FNAC of the suspected lesion in the thyroglossal duct cyst, which confirmed the diagnosis of papillary carcinoma. Additionally, FNAC of the thyroid nodules revealed a coexisting multifocal papillary carcinoma in the thyroid gland, which contributed to formulating the treatment strategy, involving total thyroidectomy and central compartment nodal dissection.

Nodal metastases are reported in approximately 10% - 11.3% of cases in the literature [4]. In our series, two out of three cases exhibited nodal metastases on final histopathology, with evidence of extra-cystic disease involving adjacent soft tissue. Distant metastases, though rare, have been documented in limited case series. For example, lung metastases were reported in one out of 14 cases in the literature [2]. 

In our series, all three patients remain disease-free, with follow-up periods ranging from 1 to 12 years.

Treatment strategies

The Sistrunk procedure is considered the gold standard treatment for thyroglossal duct cysts. As a stand-alone procedure, it involves the excision of the thyroglossal duct cyst, the central portion of the body of the hyoid bone, and a core of tissue around the thyroglossal tract, extending up to the oral cavity at the foramen cecum. Ellis and van Nostrand demonstrated the close anatomical connection between the thyroglossal tract and the hyoid bone, rendering the Sistrunk procedure the only definitive method of completely removing all the remnants of the thyroglossal duct and reducing the recurrence of cysts and fistulae following surgery [17]. It is well documented that recurrence of thyroglossal duct cysts often results from failure to remove the central portion of the hyoid bone during surgery.

For patients with high-risk features, such as papillary carcinoma within the thyroglossal duct cyst or concurrent disease in the thyroid gland, total thyroidectomy is indicated. In cases with nodal metastasis in the central compartment, nodal dissection should be considered. RAI ablation therapy should be determined based on final histopathology and RAI scan findings, in consultation with a multidisciplinary team with the focus centered on the principles of the treatment of papillary carcinoma of the thyroid gland.

In the literature, 73% of the cases of papillary carcinoma in thyroglossal duct cysts were diagnosed after a Sistrunk procedure [18]. When an unexpected diagnosis of papillary carcinoma is made postoperatively, the decision of whether to proceed with total thyroidectomy and adjuvant radioactive iodine therapy becomes crucial. If a preoperative US scan does not suggest thyroid involvement, other factors should guide the decision-making process. These factors include male sex, age > 45 years, size > 4 cm, lymph node metastases, past history of radiation to the neck region, involvement of the cyst wall on histology, palpable lymph nodes, nodular thyroid findings, and evidence of a cold nodule on thyroid scan. As the coincidence of papillary carcinoma in the thyroid gland is reported in 43-73% of cases, total thyroidectomy followed by RAI ablation and TSH suppression is recommended in high-risk patients. 

Neck nodes are managed based on the principles of thyroid malignancy. In case of gross spread to neck nodes, whether in the central or lateral compartment, appropriate nodal dissection should be performed during surgery. 

In our case series, the first two patients had preoperative FNAC results that confirmed papillary carcinoma in both the thyroglossal duct cyst and the thyroid gland nodules. One patient had extensive disease beyond the cyst wall, while the other had limited disease confined to the cyst and the thyroid nodules. The third patient was diagnosed with papillary carcinoma on histopathology of the thyroglossal duct cyst. He underwent thyroidectomy as a staged procedure, revealing microscopic foci of papillary carcinoma in the thyroid gland postoperatively. All three patients received RAI ablation and were found to be disease-free on follow-up, exhibiting low thyroglobulin levels during the follow-up period ranging from 1 to 12 years. 

## Conclusions

The evaluation of a thyroglossal duct cyst should include a thorough US scan to assess for any mural nodule. Although papillary carcinoma is rare, it can be suspected based on US findings. Preoperative US-guided FNAC from a mural nodule can help confirm the diagnosis of papillary carcinoma. A comprehensive examination of the thyroid gland for any suspicious nodules, as well as lymph nodes in the draining area, is essential. FNAC may further confirm the diagnosis in the thyroid gland and lymph nodes. The management of these cases remains controversial due to the limited number of reported instances, highlighting the importance of a multidisciplinary approach and the need to tailor treatment to each individual case.

The Sistrunk procedure is generally sufficient for most patients when the disease is confined to the thyroglossal duct cyst. However, additional management strategies such as total thyroidectomy, central compartment nodal dissection, and adjuvant RAI ablation may be considered for high-risk patients. It depends on various factors including the status of the thyroid gland, tumor size, histopathological findings, patient age, and the presence of nodal metastasis. Papillary carcinoma arising from a thyroglossal duct cyst generally carries an excellent prognosis.
